# Correlates of attempting to quit smoking among adults in Bangladesh

**DOI:** 10.1016/j.abrep.2018.04.002

**Published:** 2018-04-26

**Authors:** Shariful Hakim, Muhammad Abdul Baker Chowdhury, Md Jamal Uddin

**Affiliations:** a Department of Statistics, Shahjalal University of Science & Technology, Sylhet 3114, Bangladesh; b Department of Emergency Medicine, University of Florida College of Medicine, Gainesville, 32610, FL, USA

**Keywords:** Smoking, Cessation, Quit attempt, GATS, Bangladesh

## Abstract

**Background:**

Quit attempts are very essential in population-based smoking cessation. Little is known about the correlates of making a quit attempt of smoking in Bangladesh. We aimed to examine correlates of making a quit attempt of smoking among adults in Bangladesh.

**Methods:**

We used data from the 2009 Global Adult Tobacco Survey, Bangladesh. A total of 2217 adult current smokers (2141 males and 76 females) aged 15 years and older who participated in the survey were included. We compared socio-demographic, behavioral, motivational, knowledge and attitudes towards smoking, quitting methods utilized, use of social media to quit smoking, and environmental characteristics of current smokers who made an attempt to quit with those who made no quit attempt during the previous 12 months of the survey. We applied multivariable logistic regression models for analyzing the data.

**Results:**

Among the 2217 current smokers, 1058 (47.72%) made attempt to quit. We found respondents who smoked their first cigarette within 6 to 30 min of waking up were more likely to make an attempt to quit than those who smoked their first cigarette within 5 min of waking. Moreover, among daily current smokers who smoked 10–19 manufactured cigarettes per day were less likely to make a quit attempt. We also found intention to quit smoking, smoking rules inside the home, and exposure to anti-smoking advertisements as significant correlates of making a quit attempt of smoking among adults in Bangladesh.

**Conclusions:**

Policymakers should consider our findings when implementing tobacco control programs in Bangladesh.

## Highlights

• Quit attempts are very essential in population-based smoking cessation.
• Among the 2233 current smokers, 1058 individuals attempted to quit smoking.
• Smokers who don't smoke quickly after waking were more likely to make quit attempt.
• Smokers who lived in a smoke-free home were also more likely to make quit attempt.

## 1 Introduction

The tobacco epidemic is bigger than most other public health disasters the world has ever confronted (Mia et al., 2017). Nearly 6 million people have killed annually due to tobacco use (Jha, 2011). Unless proper steps are taken by 2030, tobacco will kill >8 million people per year globally (World Health Organization, 2017a; Hakim, Chowdhury, & Uddin, 2017). Although smoking rate is decreasing in most developed countries, it is increasing in developing countries including Bangladesh (Giovino et al., 2012). Because of the rapid rise of smoking in developing countries, by 2030, 7 million deaths will occur annually in these countries (Abdullah & Husten, 2004). Countries in Asia, especially, South East Asia (SEA) region, are responsive to smoking epidemic (Rao, Aslam, Zaheer, & Shafique, 2014). Approximately, 400 million tobacco users live in this region, which results in 1.2 million deaths annually (Sreeramareddy, Pradhan, Mir, & Sin, 2014). Therefore, increasing smoking cessation can have a substantial effect in reducing the tobacco-attributable deaths (Abdullah & Husten, 2004).

Bangladesh is one of the ten heaviest smoking countries in the world (Abdullah, Driezen, Quah, Nargis, & Fong, 2015). Bangladesh has a high smoking prevalence of 23.0% of adults who smoke which approximates to 21.9 million adults currently smoke tobacco (World Health Organization, 2017b; Hakim et al., 2017). The general smoking prevalence increased from 20.9% in 2004–05 to 22.0% in 2010 (Abdullah et al., 2015). Moreover, Bangladesh is one of the 15 countries in the world that have a greater burden of tobacco-associated illness (World Health Organization, 2017b; Hakim et al., 2017). In 2004, World Health Organization (WHO) showed that tobacco use was responsible for nearly 57,000 deaths and 1.2 million tobacco-attributable illness annually in Bangladesh (Nargis et al., 2015). Another study conducted in Bangladesh using 2010 data observed that smoking was responsible for 42,000 deaths of men (aged 25 to 69 years) (Alam et al., 2013). This study also showed that each smoker waste average 7 years of life due to smoking. Because of the high rate of tobacco-induced deaths, the health and economic burden are increasing rapidly (Nargis et al., 2015). To tackle this epidemic, there is crying need to reduce the use of tobacco, which will need preventing initiation of tobacco use and encouraging smoking cessation among smokers (Abdullah et al., 2015).

Several previous studies determined the correlates of attempts to quit smoking and smoking habit in Bangladesh. Flora, Kabir, and Moni (2017) identified the social correlates of intention to quit and quitting attempt of smoking in Bangladesh by gender and place of residence (Flora et al., 2017). This study found that intention to quit smoking was influenced by education, age at starting smoking, type of smokers and number of smoker friends and attempt to quit smoking associated with type of smokers and number of smoker friends. Driezen, Abdullah, Quah, Nargis, and Fong (2016) identified the determinants of intention to quit smoking among adults in Bangladesh (Driezen et al., 2016). In accordance with this study, intention to quit smoking associated with area of residence, number of cigarettes/bidis smoked per day, attempting to quit in the past year, visiting a doctor in last year, having children at home, perceiving health benefit from quitting, worrying about the health consequences of smoking, knowledge of second-hand smoke (SHS), enjoying smoking, and workplace smoking policy Flora, Mascie-Taylor, Rahman, and Akter (2012) examined the effect of parental smoking on adult Bangladeshis smoking habit (Flora et al., 2012). This study showed that non-smoker parents had higher chance of having nonsmoker offspring. However, the studies that examined the factors that are associated with quit attempt have been limited to specific populations such as young/adolescents (Backinger, Fagan, Matthews, & Grana, 2003; Fagan et al., 2007), health center and/or continually-ill patients (Bock, Becker, Partridge, & Niaura, 2007; Herlitz, Bengtson, Hjalmarson, & Karlson, 1995), specific ethnic background (Kahende, Malarcher, Teplinskaya, & Asman, 2011; King, Polednak, Bendel, Vilsaint, & Nahata, 2004), homeless population (Baggett, Lebrun-Harris, & Rigotti, 2013; Wrighting, Businelle, Kendzor, LeBlanc, & Reitzel, 2016), prisoners (Frank et al., 2017; Makris, Gourgoulianis, & Hatzoglou, 2012). There are a few studies that identified the correlates of making quit attempt of smoking in general populations (Abdullah et al., 2015; Ayo-Yusuf & Szymanski, 2010; Kaleta et al., 2012). For example, in a recent study in Bangladesh, making quit attempt was associated with residential areas outside Dhaka, being aged 40 or older, having a monthly income of above BDT10,000 (US$126) versus below BDT 5000 (US$63), intention to quit sometime in the future (Abdullah et al., 2015). In another study in Poland, smokers were more likely to attempt to stop if they were aged 60 years or older, had a high educational qualification, were aware of the harmful effect of smoking (Kaleta et al., 2012). Moreover, in a study in South African population, female gender, older age, having tertiary education, living in smoke-free homes, smoke >20 cigarettes per day, or having alcohol dependence in the past were significantly associated with making a quit attempt (Ayo-Yusuf & Szymanski, 2010).

Quitting smoking is a continuous process and that may involve many failed quit attempts before ultimately succeeding (Larabie 2005; Hakim et al., 2017). Therefore, quit attempts are very essential in population-based smoking cessation. In U.S.A, about 37% of all smokers have attempted to quit one to two times, 19% have made three to five attempts and 8% have tried to quit six or more times in their lifetime (Clark, Kviz, Crittenden, & Warnecke, 1998). Seventy percent of former smokers reported that they have made one to two attempts before stopping smoking in U.S.A (Clark et al., 1998). With a view to rising the odds of quitting smoking, it is critical to promote an attempt at cessation in smokers who might otherwise not try (Hakim et al., 2017). Therefore, our objective was to identify the factors that are associated with making a quit attempt of smoking using a large representative sample from a cross-sectional national survey of Bangladesh.

## 2 Methods

### 2.1 Data source

We used available nationally representative data from the 2009 Global Adult Tobacco Survey (GATS), Bangladesh (“CDC - GTSSData”, n.d.). GATS is a standardized, cross-sectional, and nationally representative household survey of adults (15 years of age or older). In Bangladesh, the National Institute of Preventive and Social Medicine (NIPSOM) conducted GATS in 2009 in collaboration with the National Institute of Population Research and Training (NIPORT), and the Bangladesh Bureau of Statistics (BBS). Moreover, the Centers for Disease Control and Prevention (CDC), USA, and the World Health Organization provided technical supports.

#### 2.1.1 Sample design

**Fig. 1:**
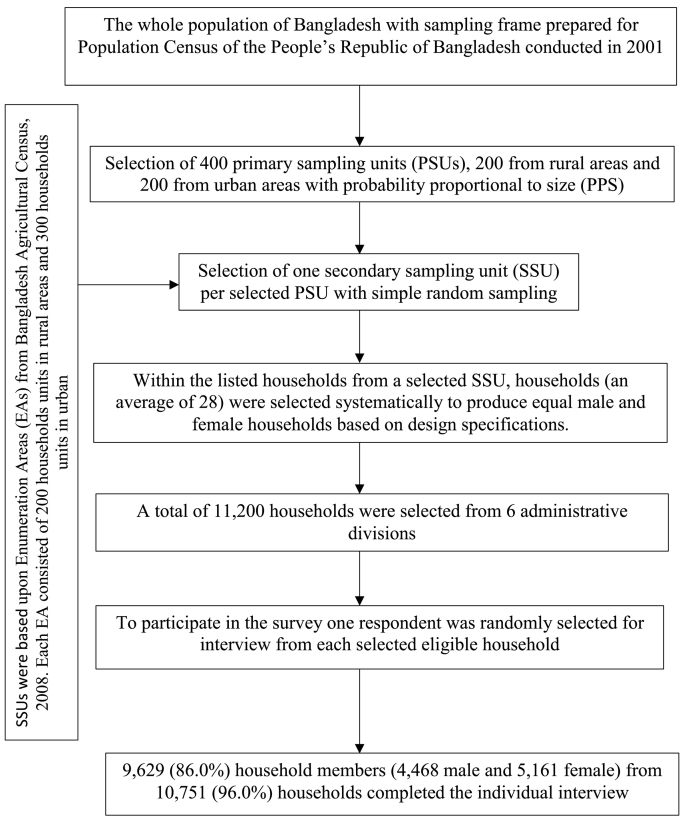
Study design of Global Adult Tobacco Survey Bangladesh, 2009.

The sample was drawn using a three-stage stratified cluster sampling. In the first stage, 400 primary sampling units (PSUs) were selected using probability proportional to size (PPS) sampling. In the second stage, a secondary sampling unit (SSU) was selected from per PSU using simple random sampling (SRS). At the third stage, an average of 28 households was selected from each SSU. With this design, 11,200 households were selected. Among the selected households, 10,050 persons were found to be eligible for the single interview. Out of 10,050 households, 9629 individuals completed the interview successfully with a response rate of 93.6%. The sampling procedure and the study design is presented in Fig. 1**.** The detailed survey procedure, study method, questionnaires are available in elsewhere (“World Health Organization, Global Adult Tobacco Survey Bangladesh Report 2009”, n.d.).

### 2.2 Measures

#### 2.2.1 Outcome variable

**Fig. 2:**
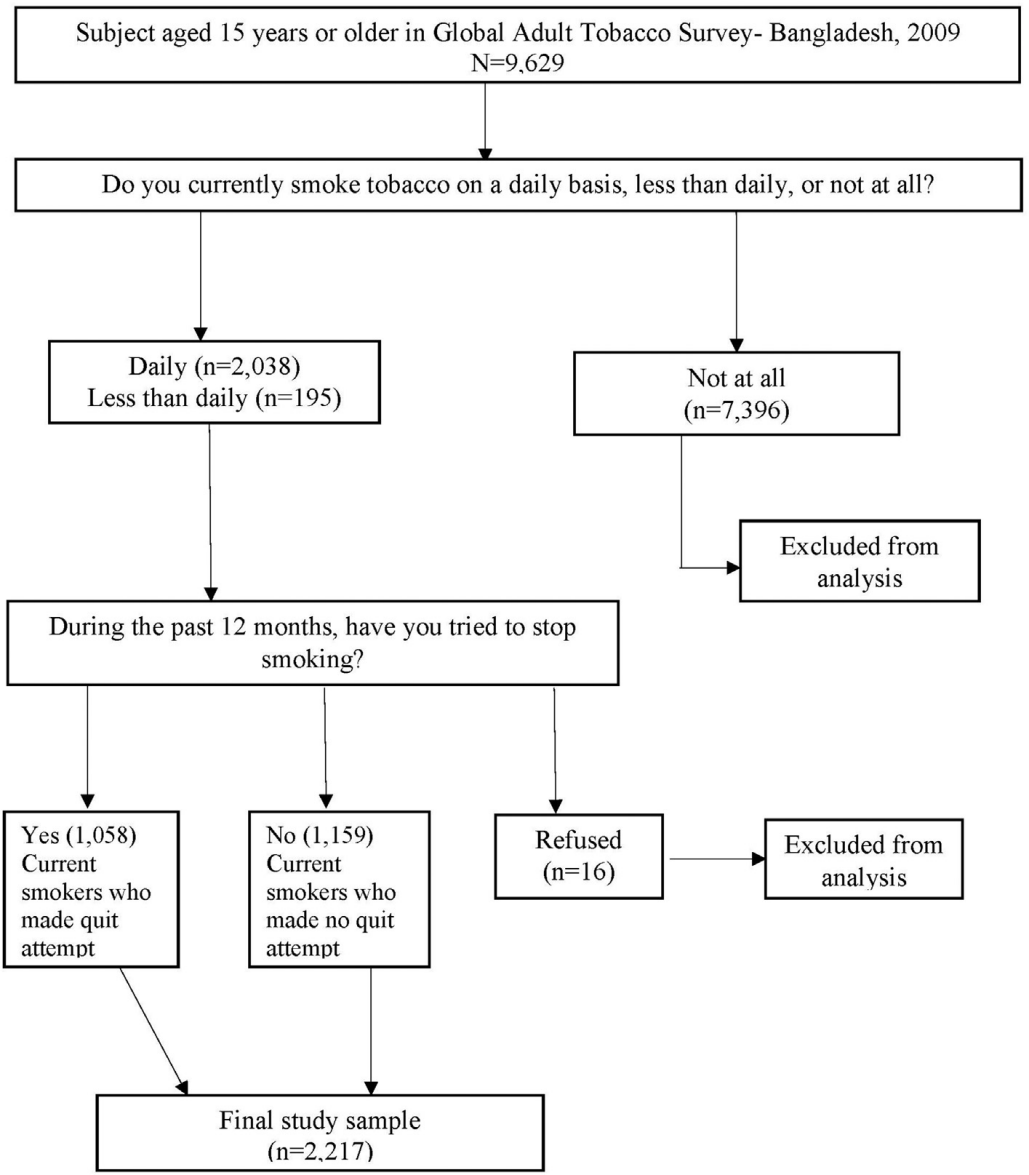
Survey screening process used to select current smokers who made any quit attempt and who made no quit attempt.

We compared the current smokers who made a quit attempt with the current smokers who made no quit attempt in the past 12 months of the survey. Respondents were asked, “Do you currently smoke tobacco on a daily basis, less than daily, or not at all?” Those who responded “not at all” were excluded from analysis. Those who responded “daily” and “less than daily”, were current smokers. Current smokers were asked, “During the past 12 months, have you tried to stop smoking?” Response options were “yes”, “no”, or “refused”. Those who refused to answer were excluded from analysis. The screening process used to select who made a quit attempt and who made no quit attempt is illustrated in Fig. 2.

#### 2.2.2 Potential factors

Six socio-demographic characteristics such as age (categorized as 15–24, 25–34, 35–44, 45–54, and 55 years or older), sex (male, female), place of residence (urban, rural), occupation (employed, business, farmers, laborers, and unemployed), education (high, moderate, low and no formal education), wealth index (highest, high, middle, low, and lowest) was used in this study. The wealth index was created using principal component analysis (“World Health Organization, Global Adult Tobacco Survey Bangladesh Report 2009”, n.d.).

Behavioral characteristics included smoked tobacco used status (daily, occasional), use of smokeless tobacco products (yes, no), age at initiation of smoking (<15 years, 15–25 years, and > 25 years), number of manufactured cigarettes smoked per day (no manufactured cigarettes, 1–9, 10–19, 20+), time to first cigarette after waking up (within 5 min, 6 to 30 min, 31 to 60 min, >60 min). Motivational factor included current smoker intention to quit (quit within the next month, thinking within the next 12 months, quit someday, but not next 12 months, and not interested in quitting). Knowledge and attitudes towards smoking indicated, belief that smoking causes serious illness (yes, no), belief that cigarettes are addictive (yes, no), and opinion about increasing taxes on tobacco products (favor, not favor). Environmental characteristics indicated smoking rules inside home (allowed, not allowed, but with exceptions, never allowed, and no rules), smoking policy at work place (allowed, not allowed, but with exceptions, never allowed, and no rules). Quitting methods utilized included advised to quit smoking (yes, no). Use of social media to quit smoking indicated exposure to anti-smoking advertisements (yes, no).

#### 2.2.3 Data analysis

We compared the percentage of potential factors between current smokers who have attempted to quit and current smokers who have not attempted to quit during the past 12 months of the survey using the Chi-square test. Binary logistic regression analysis was used to identify the factors that are associated with making a quit attempt of smoking. We evaluated for co-linearity using variance inflation factor (VIF) with a cutoff 4.0. We obtain all estimates and confidence intervals (CI) from weighted data, and the multistage stratified cluster sampling design was accounted for variance estimations.

#### 2.2.4 Variable selection and model diagnostics

Logistic regression model was formed using forward selection procedure. First, we formed a null model with no predictors. Then, the first potential factor considered for entry into the model was the one, which was the most significant (with the smallest *p*-value). After the first factor was entered, the potential factor not in the model that has the smallest p-value was considered next. The procedure was repeated until no (additional) effects met the 5% significance level for entry into the model. We calculated Akaike Information Criteria (AIC) in each step (Table S1). To assess the overall fit of the final model, we used Pearson Chi-square and Hosmer-Lemeshow goodness of fit statistic (Table S2). To reflect the predictive accuracy of the final model, we used area under the curve (AUC) of receiver operating characteristic curve (ROC) (Fig. S1).

Statistical software SPSS (version 21.0) and SAS version 9.4 (SAS Institute Inc., Cary, NC) were used for data management and analysis.

## 3 Results

### 3.1 Study subjects

Among the 9629 adults, (aged 15 years or older) 2233 were current smokers, and 7396 were nonsmokers. Therefore, the 7396 individuals were excluded from analysis. Among the current smokers, 1159 individuals made no quit attempt, and 1058 individuals attempted to quit smoking during the past 12 months of the survey. Thus, the 1058 current smokers who attempted to quit and 1159 current smokers who made no attempt to quit were the final study subjects (Fig. 2).

### 3.2 Bivariate analysis

The bivariate comparison of study variables by making attempt to quit status are illustrated in Table 1. Considering socio-demographic variables, among current smokers, who had attempted to quit, about 50.6% were aged between 25 and 34 years old, 48.2% were male, 52.1% lived in urban areas, 62.5% were employed, 56.8% had high level of education, and 55.2% had highest wealth index.

**Table 1:** Bivariate comparison of selected characteristics by making attempt to quit smoking status: Global Adult Tobacco Survey, Bangladesh-2009.

Variables	Made no attempt to quit, n (%)	Made attempt to quit, n (%)	*p*-Value
Age (yr)			0.228
15–24	124 (53.7)	107 (46.3)	
25–34	288 (49.4)	295 (50.6)	
35–44	336 (52.3)	306 (47.7)	
45–54	206 (51.2)	196 (48.8)	
55 years and above	205 (57.1)	154 (42.9)	
Sex			0.030
Male	1110 (51.8)	1031 (48.2)	
Female	49 (64.5)	27 (35.5)	
Place of residence			<0.001
Urban	503 (47.9)	547 (52.1)	
Rural	656 (56.2)	511 (43.8)	
Occupation			<0.001
Employed	97 (37.5)	162 (62.5)	
Business	244 (51.6)	229 (48.4)	
Farmers	232 (53.1)	205 (46.9)	
Laborers	488 (55.2)	396 (44.8)	
Unemployed	98 (59.8)	66 (40.2)	
Education			0.013
High	38 (43.2)	50 (56.8)	
Moderate	242 (47.4)	269 (52.6)	
low	319 (53.5)	277 (46.5)	
No education	560 (54.8)	462 (45.2)	
Wealth index			0.001
Highest	134 (44.8)	165 (55.2)	
High	209 (48.4)	223 (51.6)	
Middle	211 (52.8)	189 (47.3)	
Low	301 (53.1)	266 (46.9)	
Lowest	304 (58.6)	215 (41.4)	

Behavioral characteristics			
Smoked tobacco use status			0.036
Daily user	1071 (53)	951 (47)	
Occasional user	88 (45.1)	107 (54.9)	
Use of smokeless tobacco products			0.251
Yes	342 (50.4)	336 (49.6)	
No	817 (53.1)	722 (46.9)	
Age at initiation of smoking			0.055
> 25	65 (43.3)	85 (56.7)	
15–25	782 (53.2)	688 (46.8)	
< 15	193 (54.4)	162 (45.6)	
Missing	119 (49.2)	123 (50.8)	
Time to first cigarette smoking after waking up			<0.001
>60 min	268 (44.8)	330 (55.2)	
31 to 60 min	265 (52)	245 (48)	
6 to 30 min	388 (57.4)	288 (42.6)	
Within 5 min	147 (63.1)	86 (36.9)	
Missing	91 (45.5)	109 (54.5)	
No. of manufactured cigarettes smoked per day			<0.001
20+	90 (54.2)	76 (45.8)	
10–19	261 (57)	197 (43)	
1–9	290 (42.9)	386 (57.1)	
No manufactured cigarette	417 (59.6)	283 (40.4)	
Missing	101 (46.5)	116 (53.5)	

Motivational characteristic			
Intention to quit smoking			<0.001
Quit within the next month	362 (55.2)	294 (44.8)	
Thinking within the next 12 months	130 (31.3)	285 (68.7)	
Quit someday, but not in the next 12 months	114 (28.5)	286 (71.5)	
Not interested in quitting	394 (83.3)	79 (16.5)	
Missing	159 (58.2)	114 (41.8)	

Knowledge and attitudes towards smoking			
Belief that smoking causes serious illness			0.036
Yes	1121 (52.3)	1021 (47.7)	
No	16 (36.4)	28 (63.6)	
Missing	22 (71)	9 (29)	
Belief that cigarettes are addictive			0.16
Yes	1068 (52.7)	960 (47.3)	
No	79 (47)	89 (53)	
Missing	12 (57.2)	9 (42.8)	
Opinion about increasing taxes on tobacco products			0.001
Favor	628 (49.7)	635 (50.3)	
Not favor	387 (58.5)	232 (41.5)	
Missing	204 (51.6)	191 (48.4)	

Environmental characteristics			
Smoking rules inside home			<0.001
Allowed	488 (62.6)	291 (37.4)	
Not allowed, but with exceptions	119 (37.3)	200 (62.7)	
Never allowed	146 (38)	238 (62)	
No rules	404 (55.1)	329 (44.9)	
Missing	2 (100)		
Smoking policy at workplace			<0.001
Allowed	133 (48.7)	140 (51.3)	
Not allowed, but with exceptions	32 (29.4)	77 (70.6)	
Never allowed	67 (32.7)	138 (67.3)	
No rules	134 (55.4)	108 (44.6)	
Missing	793 (57.1)	595 (42.9)	
Quitting methods utilized			
Advised to quit smoking			0.026
Yes	186 (39.5)	285 (60.5)	
No	17 (60.7)	11 (39.3)	
Missing	956 (55.6)	762 (44.4)	

Use of social media to quit smoking			
Exposure to anti-smoking advertisements			<0.001
Yes	927 (49.8)	933 (50.2)	
No	332 (65)	125 (35)	

For behavioral variables, among current smokers who had attempted to quit, 49.6% used smokeless tobacco, 54.9% were occasional tobacco user, 56.7% started smoking after 25 years old, 55.2% first smoked >60 min of waking up, 57.1% smoked manufactured cigarette from 1 to 9. For the motivational characteristic, among current smokers who made quit attempt, 68.7% were thinking to quit within the next 12 months.

Considering the knowledge and attitudes towards smoking, among current smokers who had attempted to quit, about 63.6% did not believe that smoking cause's serious illness, 53.0% did not believe that cigarettes are addictive, and 50.3% were in favor of increasing taxes on tobacco products.

Considering the environmental characteristics, among the current smokers who made a quit attempt, smoking was not allowed, but with exceptions inside their homes (62.7%), and 70.6% of smokers workplace smoking was not allowed, but with exceptions.

Considering the quitting methods utilized, 60.5% were advised to quit smoking in current smokers who had attempted to quit. For the use of social media to quit smoking, among current smokers who had attempted to quit, 50.2% were exposed to antismoking advertisements.

### 3.3 Multivariable analysis

The summary results of the logistic regression model for making a quit attempt are shown in Table 2**.** With respect to behavioral characteristics, respondents who smoked their first cigarette within 6 to 30 min of waking up were 1.44 times more likely (Odds ratio (OR) = 1.44, 95% confidence interval (CI) = 0.87–2.36) to make an attempt to quit than who smoked their first cigarette within 5 min of waking, and who smoked 10–19 manufactured cigarettes per day were less likely (OR = 0.65, 95% CI: 0.43–0.98) to make a quit attempt than those who smoked nine or less manufactured cigarettes per day. With respect to motivational characteristic, smokers who will quit someday, but not in the next 12 months were 13.74 times more likely (OR = 13.74, 95% CI: 8.02–23.53) to make a quit attempt than who were not interested in quitting. With respect to environmental characteristics, among those smokers where smoking in the house was never allowed were 2.58 times more likely (OR = 2.58, 95% CI: 1.71–3.92) to make an attempt to quit smoking. With respect to the use of social media to quit smoking, smokers who were exposed to antismoking advertisements on media were 1.55 times more likely (OR = 1.55, 95% CI: 0.94–2.54) to make a quit attempt than who were not exposed to antismoking advertisements.

**Table 2:** Correlates of making an attempt to quit: odds ratio and 95% confidence intervals from multivariable logistic regression model.

Effect	OR (95% CI)	*p*-Value
Time to first cigarette smoking after waking up		
>60 min	1.09 (0.61, 1.97)	0.768
31to 60 min	1.29 (0.75, 2.22)	0.362
6 to 30 min	1.44 (0.87, 2.36)	0.154
Within 5 min ®	1.00	
No. of manufactured cigarettes smoked per day		
20+	1.51 (0.79, 2.86)	0.211
10–19	0.65 (0.43, 0.98)	0.041⁎
No manufactured cigarette	1.01 (0.65, 1.56)	0.959
1–9 ®	1.00	
Intention to quit smoking		
Quit within the next month	3.93 (2.46, 6.3)	<0.0001⁎
Thinking within the next 12 months	11.13 (6.54, 18.94)	<0.0001⁎
Quit someday, but not in the next 12 months	13.74 (8.02, 23.53)	<0.0001⁎
Not interested in quitting	1.00	
Smoking rules inside home		
Allowed	1.25 (0.85, 1.84)	0.251
Not allowed, but exceptions	1.91 (1.16, 3.15)	0.011⁎
Never allowed	2.58 (1.71, 3.92)	<0.0001⁎
No rules ®	1.00	
Exposure to anti-smoking advertisements		
Yes	1.55 (0.94, 2.54)	0.084
No ®	1.00	

Notes: ® = Reference category.

* Indicate significant at 5% level (*p*-value < 0.05).

## 4 Discussion

We found that the correlates of making a quit attempt were time to first cigarette after waking up, number of manufactured cigarettes smoked per day, intention to quit smoking, smoking rules inside the home, and exposure to anti-smoking advertisements.

Our analyses showed that gender, age, level of education, and other socio-demographic characteristics were not associated with making quit attempt, which is in line with a previous study by Vangeli, Stapleton, Smit, Borland, and West (2011) (Vangeli et al., 2011). Consistent with previous finding (Fagan et al., 2007), we found time to first cigarette after waking up was significantly associated with making a quit attempt. In this study, smokers who do not smoke quickly after waking up were more likely to make a quit attempt. The time to first smoking after waking up is a specific measure of nicotine dependence (Ito et al., 2013). People who smoke quickly in the morning might have a greater addiction for nicotine (Muscat, Ahn, Richie, & Stellman, 2011). This addiction giving rise to thoughts for continuing smoking without making any quitting efforts (Girma, Assefa, & Deribew, 2010). The findings of our study suggest that encouraging the smokers who smoke quickly after waking up to make frequent quit attempts by increasing consciousness of the urgency of quitting.

In this study, individuals who smoked >20 manufactured cigarettes per day were more likely to make a quit attempt. An inline result was observed by the South African study in which the authors showed that smoking a higher number of cigarettes per day was associated with increased quit attempt (Ayo-Yusuf & Szymanski, 2010). This could be fact that heavier smokers have a high intention to quit, as they have a higher likelihood of experiencing the harmful effect of smoking (Ayo-Yusuf & Szymanski, 2010). On the other hand, those who smoked fewer cigarettes did not identify smoking as an instant danger to their health and were therefore not highly prompted to quit (Kim, 2014). Therefore, smoking cessation plans should be adapted to the smoker's level of cigarette consumption. This could be done by inspiring low-intensity smokers to make any quit attempt and by treating interventions for heavy smokers to cut down cigarette consumption with a view to quitting successfully.

Intention to quit smoking was significantly associated with making an attempt to quit, which is supported by Diemert, Bondy, Brown, and Manske (2013) (Diemert et al., 2013). Our study found that smokers who have any intention to quit smoking had higher chance of making a quit attempt than those who have no intention to quit. This suggests the necessity of motivating smokers to think about quitting by increasing awareness about the importance of quitting smoking through educational campaign. This should also motivate smokers to make repeated quit attempts even if that quit attempt fails to succeed (Driezen et al., 2016).

A previous study (Shopland, Anderson, & Burns, 2006) found that the smokers who lived in a smoke-free home were more likely to make a quit attempt. Similarly, this study found that smokers living in a house where smoking was not allowed had a higher chance of making a quit attempt. This may be due to the fact that smokers living in a smoke free household may induce other smokers and members of the household for banning smoking in home, which leads to change their smoking behavior (Farkas, Gilpin, Distefan, & Pierce, 1999). Our findings suggest that increased knowledge about the harmful effect of secondhand smoke and the benefits of quitting should be focused among urban as well as rural residents.

Similar to the findings by Farrelly et al. (2012), we found that the likelihood of making an attempt to quit was the highest among those who were exposed to anti-smoking advertisements in the media. Anti-smoking advertisements are a vital part of tobacco control programs which are designed to counter not only pro-tobacco influences but also expand pro-health messages (Hyland, Wakefield, Higbee, Szczypka, & Cummings, 2006). Smokers who are subjected to anti-smoking ads can perceive the detrimental effects of smoking, which might have influenced their determination to quit smoking. Therefore, with a view to promoting smoking cessation and minimizing the likelihood of initiation, we can carry on anti-smoking advertisements in media.

### 4.1 Strengths and limitations

Our study has several strengths: first, we used a nationally representative cross-sectional sample, which is unique in its inclusion of a broad range of factors. Second, we conducted extensive statistical models and assessed them using several model diagnostic tools. Finally, our final statistical model satisfied all model assumptions and has a good prediction power. There are some notable limitations of our study. First, this study is based on data that was collected about 9 years ago and the field of tobacco control has changed dramatically this decade. Second, as our study used the cross-sectional nature of the data, it does not allow us to see the changes of the characteristics over time. Third, many smokers may fail to remember or fail to report their quit attempts, which may influence our findings (Sharma & Szatkowski, 2014). Fourth, since the data were collected by self-reports of the respondents, smoking could be underreported due to the respondent's desire to provide a socially beneficial answer (Kim, 2014). Fifth, the definition of smokers and quitters was based on a single question of “Do you currently smoke tobacco on a daily basis, less than daily, or not at all? This was not only ignored the complexity of smoking behaviors but also complexity in smoking cessation behaviors. Finally, a number of psychological factors such as depressive disorders, and anxiety disorders, physiological factor, alcoholism that may also have associated with quit attempts were not included in this study as they were not available in the dataset.

## 5 Conclusions

Our study identified several correlates including time to first cigarette after waking up, number of manufactured cigarettes smoked per day, intention to quit smoking, smoking rules inside the home, and exposure to anti-smoking advertisements of making a quit attempt among Bangladeshi adult smokers. Policy makers should consider these factors when designing and implementing tobacco control strategies and programs. Our findings suggest a requirement to ensure targeted interventions for those smokers who have made no quit attempt, and those who are not interesting in quitting. Further research is needed for adult smokers followed repeatedly over a longer period in order to allow each respondent with a view to creating multiple opportunities to make a quit attempt.

## Role of funding sources

This research did not receive any specific grant from funding agencies in the public, commercial, or not-for-profit sectors.

## Contributors

**SH** searched the literature, planned the study, prepared the analytic dataset, conducted data analysis, interpretation, drafted the manuscript, and corrected it after comments from all co-authors. **MABC** helped in the data management and analysis, provided important comments on the interpretation of the results, draft manuscript, reviewed the final version of the manuscript. **MJU** supervised the overall work, helped in preparing, analyzing, and interpreting the data; provide very crucial scientific comments on the draft. The final manuscript was read and approved by the all authors.

## Conflict of interest

None.

## Appendix A Supplementary data

Supplementary material Image 1
