# Supplementary material for: Correlates of attempting to quit smoking among adults in Bangladesh
Source: Addict Behav Rep. 2018 Apr 26;8:1–7. doi: 10.1016/j.abrep.2018.04.002 (PMC6062856; doi:10.1016/j.abrep.2018.04.002)
Supplement: Supplementary file 1 — Supplementary material [file mmc1.docx]

**Table S1:** Summary of forward selection procedure for model: making an attempt to quit vs. making no attempt to quit.

| **Model** | **Effects entered** | **No. of effects in the model** | **P-value** | **AIC** |
| --- | --- | --- | --- | --- |
| 1 | Intention to quit smoking | 1 | <0.0001 | 19063777 |
| 2 | Smoking rules inside home | 2 | <0.0001 | 18656479 |
| 3 | Exposure to antismoking advertisements | 3 | 0.0004 | 18595200 |
| 4 | No. of manufactured cigarettes smoked per day | 4 | 0.0003 | 18471153 |
| 5 | Time to first cigarette after waking up | 5 | 0.0499 | 18420448 |

**Table S2:** GOF statistics for model making an attempt to quit vs. making no attempt to quit

| Criterion | Value | DF | P-value |
| --- | --- | --- | --- |
| Pearson | 318.12 | 298 | 0.11 |
| Hosmer-Lemeshow | 13.22 | 8 | 0.21 |

.
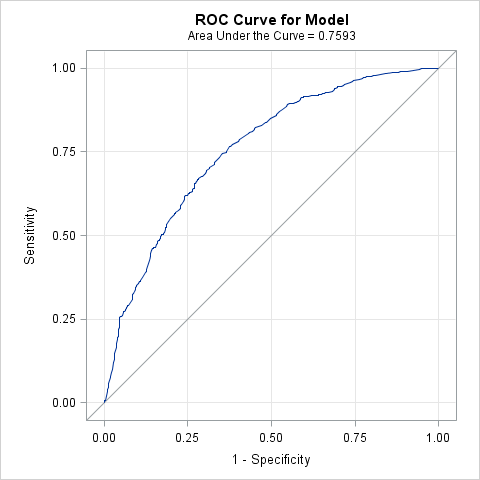


**Figure S1:** ROC curve for model making an attempt to quit vs. making no attempt to quit
